# Antibacterial activity of novel nanoparticles against persistent endodontic pathogens

**DOI:** 10.6026/973206300214807

**Published:** 2025-12-15

**Authors:** Koustav Dhara, Divya Batra, Vidya Nagabhushan, Deepinder Kaur, Rakashree Chakraborty, Priyatam Maruti Karade

**Affiliations:** 1 Department of Conservative Dentistry and Endodontics, Kalinga Institute of Dental Sciences, KIIT Deemed to be university, Bhubaneswar, Odisha, India; 2 Department of Conservative dentistry and Endodontics, National Dental College and Hospital Derabassi , Punjab, India; 3 Oral Pathologist, Pandits dental care, 15th Cross LIC colony srirampura 2nd stage, Mysore- 570023, Karnataka, India; 4 Department of Conservative Dentistry & Endodontics, Luxmi Bai Institute of Dental Sciences & Hospital, Sirhind Road, Patiala 147003, Punjab, India; 5 Department of Oral Medicine and Radiology; Maharishi Markandeshwar College of Dental Sciences and Research, Mullana, Ambala, 133207, Haryana, India; 6 Department of conservative dentistry and endodontics, Bharati Vidyapeeth, (Deemed To Be University) Dental College and Hospital, Sangli 416416, Maharashtra, India

**Keywords:** Nanoparticles, endodontic infection, *Enterococcus faecalis*, silver nanoparticles, zinc oxide, biofilm, antimicrobial activity

## Abstract

Persistent endodontic infections remain difficult to eradicate due to the resistance of biofilm-forming pathogens to conventional medicaments. Therefore, it is of interest to evaluate Ag-ZnO hybrid nanoparticles synthesized via a sol-gel method for their antimicrobial efficacy against *E. faecalis*, *C. albicans* and *A. israelii*. The nanoparticles showed strong antibacterial activity, including large inhibition zones, low MIC values (12.5-25 µg/mL) and 87.3% biofilm suppression at 100 µg/mL. Cytotoxicity testing confirmed >80% fibroblast viability at therapeutic concentrations. Ag-ZnO NPs demonstrated potent antimicrobial performance with acceptable biocompatibility, supporting their potential as an advanced intracanal medicament.

## Background:

Endodontic infections represent a complex polymicrobial condition involving the root canal system and periradicular tissues, frequently requiring root canal treatment for management [1]. Despite advances in endodontic techniques and antimicrobial protocols, treatment failures occur in approximately 10-15% of cases, primarily attributed to persistent intraradicular infections [2]. The bacterial species most commonly associated with treatment-resistant endodontic infections include *Enterococcus faecalis*, *Candida albicans*, *Actinomyces species* and *Streptococcus species* [3]. *Enterococcus faecalis*, a gram-positive facultative anaerobe, has been detected in 30-77% of failed endodontic cases [4]. This microorganism demonstrates remarkable resilience through multiple virulence factors, including adherence to dentin, biofilm formation, resistance to nutrient deprivation and survival in alkaline environments [5]. The ability of *E. faecalis* to invade dentinal tubules up to 1000 µm in depth creates anatomical sanctuaries that protect bacteria from conventional antimicrobial agents [6]. Current intracanal medicaments, including calcium hydroxide and chlorhexidine gluconate, have shown limitations in completely eradicating resistant pathogens and disrupting mature biofilms [7]. Calcium hydroxide, despite its widespread use, demonstrates inadequate antibacterial efficacy against *E. faecalis* biofilms and limited penetration into dentinal tubules [8]. These limitations have stimulated research toward novel antimicrobial strategies with enhanced efficacy and biocompatibility profiles. Nanotechnology has emerged as a promising frontier in endodontic antimicrobial therapy [9]. Metallic nanoparticles, particularly silver (Ag) and zinc oxide (ZnO) nanoparticles, have demonstrated potent broad-spectrum antimicrobial properties through multiple mechanisms including cell membrane disruption, protein denaturation, DNA damage and reactive oxygen species generation [10]. Recent studies have explored various nanoparticle formulations for endodontic applications [11, 12]. Silver nanoparticles exhibit exceptional antibacterial properties at low concentrations, while zinc oxide nanoparticles demonstrate excellent biocompatibility and osseointegration potential [13]. The synergistic combination of silver and zinc oxide in hybrid nanoparticle systems may potentially enhance antimicrobial efficacy while minimizing cytotoxicity concerns [14]. Despite promising preliminary findings, there remains a paucity of comprehensive data evaluating hybrid metallic nanoparticle formulations against the spectrum of persistent endodontic pathogens, particularly regarding biofilm eradication and biocompatibility profiles. Therefore, it is of interest to synthesize and characterize silver-zinc oxide hybrid nanoparticles and evaluate their antibacterial activity against persistent endodontic pathogens (*E. faecalis*, *C. albicans* and *A. israelii*) in comparison with conventional endodontic antimicrobials, while assessing their cytotoxic effects on human periodontal ligament fibroblasts.

## Materials and Methods:

## Synthesis of Silver-Zinc Oxide Hybrid Nanoparticles:

Ag-ZnO hybrid nanoparticles were synthesized using a modified sol-gel method. Silver nitrate (AgNO_3_, 99.9% purity, Sigma-Aldrich, USA) and zinc acetate dihydrate (Zn(CH_3_COO)_2_.2H_2_O, 99% purity, Merck, Germany) were used as precursors at a molar ratio of 1:3 (Ag:Zn). Briefly, 0.5 M zinc acetate solution was prepared in absolute ethanol under continuous magnetic stirring at 60°C. Subsequently, 0.167 M silver nitrate solution was added dropwise. Sodium hydroxide (2 M) was added as a reducing and precipitating agent to maintain pH at 11-12. The reaction mixture was stirred continuously for 3 hours at 60°C, then aged for 24 hours at room temperature. The precipitate was collected by centrifugation at 8000 rpm for 15 minutes, washed five times with deionized water and ethanol and dried at 80°C for 12 hours. The dried powder was calcined at 400°C for 2 hours in a muffle furnace.

## Characterization of nanoparticles:

Morphological analysis was performed using transmission electron microscopy (TEM, JEOL JEM-2100, Japan) at 200 kV accelerating voltage. Crystalline structure was determined by X-ray diffraction (XRD, Rigaku MiniFlex 600, Japan) with Cu-Kα radiation (λ=1.54 Å) over a 20 range of 20-80°. Particle size distribution and zeta potential were measured using dynamic light scattering (DLS, Malvern Zetasizer Nano ZS, UK). Functional groups were identified by Fourier-transform infrared spectroscopy (FTIR, Perkin Elmer Spectrum 100, USA) in the range of 400-4000 cm^-1^.

## Bacterial strains and culture conditions:

Three reference strains were obtained from the American Type Culture Collection (ATCC): *Enterococcus faecalis* ATCC 29212, *Candida albicans* ATCC 10231 and Actinomyces israelii ATCC 10049. Bacterial cultures were maintained on Brain Heart Infusion (BHI) agar at 37°C under appropriate atmospheric conditions (aerobic for *E. faecalis* and *C. albicans*, anaerobic for *A. israelii* using anaerobic gas packs).

## Agar diffusion assay:

Antibacterial activity was assessed using the Kirby-Bauer disk diffusion method. Bacterial suspensions were prepared to match 0.5 McFarland standard (1.5 x 10^8^ CFU/mL) and spread uniformly on Mueller-Hinton agar plates. Sterile filter paper disks (6 mm diameter) were impregnated with 20 µL of test solutions: Ag-ZnO NPs (100 µg/mL), calcium hydroxide paste (Ca(OH)_2_, Metapex, Meta Biomed, Korea), 2% chlorhexidine gluconate (CHX, Sigma-Aldrich, USA) and sterile distilled water as negative control. Plates were incubated at 37°C for 24 hours and zones of inhibition were measured in millimeters using digital calipers. All experiments were performed in quadruplicate.

## Minimum inhibitory concentration (MIC) and minimum bactericidal concentration (MBC):

MIC was determined using the broth microdilution method in 96-well microplates according to Clinical and Laboratory Standards Institute guidelines. Serial two-fold dilutions of Ag-ZnO NPs ranging from 200 to 0.78 µg/mL were prepared in BHI broth. Bacterial suspensions (1 x 10^6^ CFU/mL) were added to each well and plates were incubated at 37°C for 24 hours. MIC was defined as the lowest concentration showing no visible bacterial growth. For MBC determination, 10 µL aliquots from wells showing no growth were subcultured on BHI agar and incubated for 24 hours. MBC was defined as the lowest concentration resulting in ≥99.9% bacterial killing.

## Biofilm formation and inhibition assay:

Biofilm formation was performed in 96-well flat-bottom microplates. Bacterial suspensions (1 x 10^6^ CFU/mL) in BHI broth supplemented with 1% glucose were incubated at 37°C for 48 hours with media replacement at 24 hours. After biofilm formation, planktonic cells were removed and biofilms were treated with various concentrations of Ag-ZnO NPs (25, 50, 100 µg/mL) for 24 hours. Biofilm biomass was quantified using crystal violet staining. Briefly, biofilms were washed, fixed with methanol, stained with 0.1% crystal violet for 15 minutes, washed and dissolved in 33% acetic acid. Absorbance was measured at 595 nm using a microplate reader (BioTek ELx800, USA). Biofilm inhibition percentage was calculated relative to untreated controls.

## Cytotoxicity assessment:

Human periodontal ligament fibroblasts (HPDLFs) were obtained from healthy extracted third molars with informed consent and institutional ethical approval (Protocol No. IRB-2023-045). Cells were cultured in Dulbecco's Modified Eagle Medium supplemented with 10% fetal bovine serum and 1% penicillin-streptomycin at 37°C in 5% CO_2_. Cell viability was assessed using the MTT (3-(4,5-dimethylthiazol- 2-yl)-2,5-diphenyltetrazolium bromide) assay. HPDLFs were seeded in 96-well plates at 1 x 10^4^ cells/well and treated with various concentrations of Ag-ZnO NPs (12.5-200 µg/mL) for 24 hours. MTT solution (5 mg/mL) was added and formazan crystals were dissolved in dimethyl sulfoxide. Absorbance was measured at 570 nm and cell viability was calculated as percentage of untreated controls.

## Statistical analysis:

All experiments were performed in triplicate or quadruplicate and data were expressed as mean ± standard deviation (SD). Statistical analysis was conducted using SPSS version 26.0 (IBM Corporation, USA). Normality was assessed using the Shapiro-Wilk test. One-way analysis of variance (ANOVA) followed by Tukey's post-hoc test was employed for multiple group comparisons. Independent t-tests were used for two-group comparisons. Statistical significance was set at p<0.05.

## Results:

TEM analysis revealed spherical to quasi-spherical morphology with relatively uniform size distribution. The mean particle size measured by TEM was 42.3 ± 5.8 nm. DLS analysis showed hydrodynamic diameter of 68.7 ± 12.4 nm with polydispersity index of 0.24, indicating moderate monodispersity. Zeta potential was measured at -28.6 ± 3.2 mV, suggesting good colloidal stability. XRD analysis confirmed the crystalline structure with characteristic peaks corresponding to both metallic silver (20 = 38.1°, 44.3°, 64.5°) and wurtzite zinc oxide (20 = 31.8°, 34.4°, 36.3°, 47.5°). FTIR spectroscopy revealed Zn-O stretching vibrations at 450-500 cm^-1^ and surface hydroxyl groups at 3400-3450 cm^-1^. Table 1 (see PDF) presents the zones of inhibition for different antimicrobial agents against the tested pathogens. Ag-ZnO NPs demonstrated significantly larger inhibition zones compared to calcium hydroxide against all tested microorganisms (p<0.001). Against *E. faecalis*, Ag-ZnO NPs showed superior activity compared to chlorhexidine (18.4 ± 1.2 mm vs. 14.6 ± 1.1 mm, p<0.01). The nanoparticles exhibited the highest activity against *C. albicans* (21.7 ± 1.5 mm), followed by *E. faecalis* and *A. israelii*. Data presented as mean ± SD (n=4). Different superscript letters indicate statistically significant differences within each column (p<0.05, one-way ANOVA followed by Tukey's post-hoc test). Table 2 (see PDF) shows the MIC and MBC values of Ag-ZnO NPs against the tested pathogens. The lowest MIC was observed against *C. albicans* (12.5 µg/mL), while *E. faecalis* and *A. israelii* showed MIC values of 25 µg/mL. The MBC values were consistently 2-fold higher than MIC values for all tested microorganisms, indicating bactericidal activity at clinically achievable concentrations. Ag-ZnO NPs demonstrated concentration-dependent biofilm inhibition against all tested pathogens (Table 3 - see PDF). At 100 µg/mL concentration, biofilm inhibition ranged from 82.4% to 87.3%. *C. albicans* biofilms showed the highest susceptibility, with 87.3 ± 4.2% inhibition at 100 µg/mL. Even at the lowest tested concentration (25 µg/mL), Ag-ZnO NPs achieved 45-52% biofilm inhibition. Calcium hydroxide demonstrated significantly lower biofilm inhibition (28.6 ± 3.8% against *E. faecalis* at equivalent concentration, p<0.001). Data presented as mean ± SD percentage inhibition (n=3). *Calcium hydroxide tested at comparable concentration (100 µg/mL active component). All Ag-ZnO NP groups showed significantly higher biofilm inhibition compared to Ca(OH)_2_ (p<0.001). MTT assay results indicated concentration-dependent cytotoxicity of Ag-ZnO NPs on HPDLFs. Cell viability remained above 80% at concentrations ≤50 µg/mL, suggesting acceptable biocompatibility at therapeutic concentrations. At 100 µg/mL, cell viability decreased to 72.4 ± 6.3%, while higher concentrations (200 µg/mL) showed 48.6 ± 5.8% viability. The therapeutic concentrations required for antibacterial activity (25-50 µg/mL based on MIC/MBC) demonstrated minimal cytotoxicity with viability >80%, indicating a favorable therapeutic index.

## Discussion:

This study demonstrated that silver-zinc oxide hybrid nanoparticles possess potent antibacterial activity against persistent endodontic pathogens, significantly outperforming conventional antimicrobial agents including calcium hydroxide and chlorhexidine. The synthesized Ag-ZnO NPs exhibited favorable physicochemical characteristics with particle size in the optimal range for antimicrobial activity and adequate colloidal stability, supporting their potential application as intracanal medicaments. The superior antibacterial efficacy of Ag-ZnO NPs can be attributed to multiple synergistic mechanisms. Silver nanoparticles exert antimicrobial effects through silver ion (Ag^+^) release, which disrupts bacterial cell membranes, interferes with DNA replication and inhibits respiratory chain enzymes [15]. Zinc oxide nanoparticles generate reactive oxygen species (ROS), causing oxidative stress and bacterial cell death [16]. The hybrid formulation likely provides additive or synergistic effects, enhancing overall antimicrobial potency while potentially reducing the required concentration of each component. The significant activity against *E. faecalis* biofilms is particularly clinically relevant, as this organism represents the predominant pathogen in persistent endodontic infections [17]. Previous studies have demonstrated the limited efficacy of calcium hydroxide against *E. faecalis*, primarily due to its inability to maintain sufficiently high pH in the presence of dentin buffering capacity and limited penetration into biofilms [18]. Our findings corroborate these observations, with calcium hydroxide showing substantially inferior antibacterial and anti-biofilm activity compared to Ag-ZnO NPs. The anti-biofilm activity of Ag-ZnO NPs observed in this study aligns with recent investigations demonstrating nanoparticle penetration into biofilm matrix and interaction with bacterial cells [19]. The small particle size (42.3 nm) likely facilitates penetration through the extracellular polymeric substance matrix, enabling direct contact with embedded bacterial cells. Furthermore, nanoparticles may disrupt biofilm architecture by interfering with quorum sensing mechanisms and extracellular matrix production [20]. The MIC and MBC values obtained in this study (12.5-50 µg/mL) are clinically achievable and compare favorably with previously reported values for metallic nanoparticles against endodontic pathogens [21, 22]. The MBC/MIC ratio of 2 indicates bactericidal rather than bacteriostatic activity, which is advantageous for eradicating persistent infections. Several studies have reported similar MIC values for silver nanoparticles against *E. faecalis*, typically ranging from 16-64 µg/mL depending on synthesis methods and particle characteristics [23]. The cytotoxicity profile represents a critical consideration for clinical translation. Our findings demonstrated acceptable biocompatibility at therapeutic concentrations, with cell viability exceeding 80% at concentrations up to 50 µg/mL. This therapeutic window suggests potential for clinical application without significant adverse effects on periradicular tissues. However, previous investigations have shown variable cytotoxicity profiles for silver-based nanoparticles depending on particle size, coating materials and cell types evaluated [24]. Surface modifications with biocompatible polymers may further enhance the safety profile while maintaining antimicrobial efficacy [25]. The enhanced activity against *C. albicans* observed in this study is noteworthy, as fungal infections contribute to approximately 3-18% of persistent endodontic cases [26]. Traditional antimicrobial protocols often lack adequate antifungal activity, necessitating additional therapeutic interventions. The broad-spectrum activity of Ag-ZnO NPs against both bacterial and fungal pathogens represents a significant advantage over conventional single-mechanism antimicrobials. Several limitations should be acknowledged. This study employed *in vitro* methodologies that may not fully replicate the complex environment of infected root canal systems, including anatomical complexity, dentin interaction and host immune responses. Future investigations should include *ex vivo* models using extracted teeth and *in vivo* animal studies to validate clinical applicability. Additionally, long-term stability, optimal delivery vehicles and potential for bacterial resistance development require comprehensive evaluation [27]. The synthesis method employed in this study (sol-gel technique) offers reproducibility and scalability advantages. However, alternative green synthesis approaches using plant extracts or biological reducing agents may enhance biocompatibility and reduce environmental impact [28]. Comparative studies evaluating different synthesis protocols would provide valuable insights into optimization strategies. Clinical implementation considerations include development of appropriate delivery systems, such as incorporation into endodontic sealers, irrigation solutions, or controlled-release vehicles.

## Conclusion:

Silver-zinc oxide hybrid nanoparticles synthesized via sol-gel method demonstrated superior antibacterial and anti-biofilm activity against persistent endodontic pathogens, including *Enterococcus faecalis*, *Candida albicans* and Actinomyces israelii, significantly outperforming conventional antimicrobial agents. The nanoparticles exhibited favorable physicochemical properties, with mean particle size of 42.3 nm, good colloidal stability and crystalline structure. The MIC values ranged from 12.5-25 µg/mL, with bactericidal activity at clinically achievable concentrations.
